# Acute maternal stress in pregnancy and schizophrenia in offspring: A cohort prospective study

**DOI:** 10.1186/1471-244X-8-71

**Published:** 2008-08-21

**Authors:** D Malaspina, C Corcoran, KR Kleinhaus, MC Perrin, S Fennig, D Nahon, Y Friedlander, S Harlap

**Affiliations:** 1 Department of Psychiatry, New York University School of Medicine, New York, NY, USA; 2 Department of Psychiatry, Columbia University, New York, NY, USA; 3 New York State Psychiatric Institute, New York, NY, USA; 4 Shalvata Mental Health Center, Ramat Gan, Israel; 5 Sackler School of Medicine, Tel Aviv University, Israel; 6 Department of Information and Evaluation, Ministry of Health, Jerusalem, Israel; 7 Braun School of Public Health, Hebrew University of Jerusalem, Israel; 8 Department of Epidemiology, Mailman School of Public Health, Columbia University, New York, NY, USA

## Abstract

Schizophrenia has been linked with intrauterine exposure to maternal stress due to bereavement, famine and major disasters. Recent evidence suggests that human vulnerability may be greatest in the first trimester of gestation and rodent experiments suggest sex specificity. We aimed to describe the consequence of an acute maternal stress, through a follow-up of offspring whose mothers were pregnant during the Arab-Israeli war of 1967. A priori, we focused on gestational month and offspring's sex.

In a pilot study linking birth records to Israel's Psychiatric Registry, we analyzed data from a cohort of 88,829 born in Jerusalem in 1964–76. Proportional hazards models were used to estimate the relative risk (RR) of schizophrenia, according to month of birth, gender and other variables, while controlling for father's age and other potential confounders. Other causes of hospitalized psychiatric morbidity (grouped together) were analyzed for comparison.

There was a raised incidence of schizophrenia for those who were in the second month of fetal life in June 1967 (RR = 2.3, 1.1–4.7), seen more in females (4.3, 1.7–10.7) than in males (1.2, 0.4–3.8). Results were not explained by secular or seasonal variations, altered birth weight or gestational age. For other conditions, RRs were increased in offspring who had been in the third month of fetal life in June 1967 (2.5, 1.2–5.2), also seen more in females (3.6, 1.3–9.7) than males (1.8, 0.6–5.2).

These findings add to a growing literature, in experimental animals and humans, attributing long term consequences for offspring of maternal gestational stress. They suggest both a sex-specificity and a relatively short gestational time-window for gestational effects on vulnerability to schizophrenia.

## Background

Stress has become frequent in developed countries, as terrorist threats, actions and their aftermaths are experienced in otherwise stable populations. Concern about consequences for offspring whose mothers were stressed during pregnancy derives from strong evidence, from experimental biology, that intrauterine stress can affect neurodevelopment, altering behaviors in animals that are thought relevant to models of cognition, aggression, anxiety and depression [1]. Prenatal stress changes the way that glucocorticoids and sex hormones regulate neurogenesis in the developing brain, e.g. in the hippocampus [2]. By modifying the fetal hypothalamic-adrenal axis (HPA) and other systems, stress in pregnancy perturbs endocrine function in animal models of diabetes, cardiovascular disease and metabolic syndrome. Recent work shows sex differences in fetal responses to maternal stress [3-6] that may be specific to the gestational age at which the stress is applied experimentally. In humans, too, there is strong evidence that maternal stress in pregnancy can lead to type 2 diabetes, hypertension and metabolic syndrome in the offspring [1]. There are added concerns regarding a contribution of intrauterine stress to human attention deficits, cognitive performance, anxiety, depression, autism and schizophrenia [7,8]. As in experimental animals, there is some evidence in humans for unequal responses of males and females to stress at different stages of intrauterine life [8].

Epidemiologic observations have linked the incidence of schizophrenia to various maternal stressors during pregnancy, including bereavement [9,10], famine [11,12], military invasion [13], flood [14] and earthquake [15]. Reduced cognitive ability [16] and autism [17] have also been linked to such stresses. Until recently, the literature has been limited by low statistical power and it has been seldom possible to separate short term psychic stressors from the long term influence of disrupted environments, diets or lifestyles that might act as chronic stressors during childhood. Moreover, possible effects of season have generally been ignored, as well as demographic variables that tend to vary among births at different seasons (e.g. parental ages and social class).

While earlier studies lacked specificity regarding the timing of stress and its character, there is emerging evidence that it is early rather than later pregnancy that is the period when maternal stress is most damaging to the fetus [10,11]. Studies in non-human primates also point to very early gestation as the most sensitive period in which intrauterine stress causes adverse outcomes [18]. It is well known from experimental teratology that organ development proceeds in the embryo and fetus in an orderly sequence; anatomical defects can be induced experimentally by radiation, teratogens and/or malnutrition only during short windows of vulnerability. The same is true of the brain [19]. Since for schizophrenia neither mechanism nor lesion is known, identification of a window of vulnerability, if confirmed in other studies, might help understand its etiology or pathogenesis.

We questioned whether we could detect any change in the incidence of schizophrenia in the Jerusalem Perinatal Study cohort [20] following the Arab-Israeli war of June 1967 (the "Six Day War"). That stressor was short-lived; anxiety would have begun on May 18 [21] and escalated during preparations for war over the next two weeks. The population of Jerusalem would have been most stressed during the 3 days of bombardment on June 5–7. The war ended with widespread relief on June 11^th ^[21,22]. Israelis suffered minimal disruption of the environment and there were no obvious toxic exposures and no famine. Thus we consider this a "natural experiment", albeit uncontrolled, in which acute anxiety was divorced from the usual consequences of war. The stress would have been of sufficiently short duration, allowing us to classify its timing by month of gestation, rather than by trimester. Others investigated effects of this war, but applied their research to the whole of Israel and only to trimesters of pregnancy; they found no significant change in the incidence of schizophrenia [23]. We also postulated a priori that effects would vary by sex of offspring and would be most obvious in families likely to be most stressed, i.e. those living nearest to the border, and those with lower social class and lower education.

## Methods

This study relies on the Jerusalem Perinatal Study, a population-based research cohort derived from all births from 1964–76 in Western (Israeli) Jerusalem. The cohort includes data from the birth certificate and additional information from interviews with mothers, depending on the year of birth. The methods are summarized elsewhere [20,24] and characteristics of the population described [20]. The file has been used for research on schizophrenia [25,26] and for other follow-up studies [27-29].

Israel has maintained a national Psychiatric Registry since 1950; it includes a summary of all admissions to psychiatric wards and day facilities. The diagnoses for individuals with psychosis have been validated [30]. The Registry has been used for epidemiologic and clinical research in schizophrenia by us [25,26,31] and others [32-34]. This study is based on data from a pilot study that we undertook to determine whether a linkage was feasible. Personnel at Israel's Ministry of Health matched the Jerusalem cohort to the Psychiatric Registry, using only the offspring's identity numbers. They defined schizophrenia broadly, depending on one or more episodes with discharge diagnoses coded as F20–F29, ie schizophrenia, schizotypal disorder, delusional disorders, non-affective psychoses and schizoaffective disorders (International Classification of Diseases, 10th Revision), hereafter called "schizophrenia". They defined "other" as individuals without schizophrenia who had any admission(s) recorded in the Registry; these would have included affective, anxiety, personality and eating disorders, and substance abuse. A date of incidence was provided, i.e. date of first event in the Registry. The file was made anonymous by removal of identifying information; because of this, and the way the file was created, we could not aggregate siblings, assess associations within families, study severity of disease or disaggregate the "other" category. Creation and use of the file was approved by institutional review boards in Israel and New York and certified as exempt from the requirement of individual informed consent.

### Statistical analysis

Offspring born in different years were followed up to different ages, so Cox proportional hazards models [35] were employed to estimate the effects of maternal stress and covariates, using the PROC PHREG methods provided by SAS (SAS Institute Inc, Cary, NC). The time to diagnosis or death was treated in days from birth; surviving individuals were censored on Jan 1^st ^1998. For analyses relying on the whole cohort (for which there were no data on gestational age) we estimated fetal age in June 1967 by counting back from the date of birth. We did this using both calendar months and "standard months" of ~30.4 days, based on the integer value of 12 times (day of birth/365.25) +1, starting from 1^st ^Jan 1964. We also conducted a sensitivity analysis, shifting the standard month by +15, and -15 days. For a sub-cohort with data on gestational age, we analyzed the data in relation to the estimated dates of conception, defined as date of onset of last menstruation + 16 days. Date of last menstruation was available for 5,100 offspring and for 6,357 others the expected date of delivery was given, from which we assumed that conception had occurred 266 days earlier.

Covariates chosen for inclusion in the proportional hazards models were those that were significant independent predictors of the incidence of schizophrenia in this cohort, and also varied by season of birth (in this cohort) or following disasters (in others). Such variables included paternal age, treated as a linear continuous variable with unknowns (0.8%) set to the mean (age 30); we treated time since marriage similarly, with unknowns (1.7%) set to the median (5 years), as previously reported [25]. Other variables treated as dichotomies or sets of mutually exclusive categories were maternal age (two categories, i.e. 30–34 and 35+ compared with a reference group of all others); male sex (versus others); the lower third of social class versus (based on the fathers' occupation [36]); calendar month (11 categories, the reference category being set arbitrarily at September); and residential area (based on census tract at birth). Results are given as adjusted relative risks of schizophrenia (RRs, i.e. hazard ratios) with 95% confidence intervals (CI), no allowance being made for multiple testing.

## Results

Of the 91,451 live born subjects, 88,829 (97.2%) were traced; linkage with the Psychiatric Registry identified 637 with schizophrenia-related diagnoses and 676 with other psychiatric disorders. As we previously reported [25] the cumulative incidence was estimated as 1.0% by age 30 and in a proportional hazards model, variables significantly predicting incidence included paternal age (relative risk (RR) = 1.39 per decade, 95% confidence limits = 1.2–1.6, p < .0001; male sex (1.4, 1.2–1.6, p = .0002); length of the parents' marriage (0.80, 0.73–0.89, p < .0001 per 5 years); and low social class (1.2, 1.0–1.5, p = .0141). Maternal age was less strongly associated with schizophrenia; compared to age < 30, the RRs associated with ages 30–34 and 35+ were 1.2 (0.94–1.5) and 1.5 (1.1–2.1, p = .0066). All the aforementioned estimates were adjusted for each other.

We considered whether there were secular or seasonal trends in incidence that might confound the interpretation of findings for any one year (data not tabulated). We found no evidence for change in incidence over the years; comparing offspring born in 1964–67 and 1968–71 with a reference group based on 1972–76 the RRs for schizophrenia were 1.0 (0.90–1.4) and 1.1 (0.9–1.4) respectively, after adjusting for parents' characteristics. There was no significant variation in schizophrenia estimated for any single year and no meaningful trend with the data reanalyzed with "epidemiologic" years set from April-March, July-June or October-September. Regarding season, while there were some variations in incidence between individual calendar months, these were not obviously seasonal; furthermore, we found no significant trend for schizophrenia by testing season as sine and cosine transformations of time in an annual cycle, together with their first harmonics. We concluded that it was appropriate to compare the cohort born to women who were pregnant in June 1967 with the entire data set, adjusting for calendar month of birth and demographic variables.

Table 1 shows the raw data assessing effects of the war. The left side of the table shows the cohorts defined by calendar month of birth, while the right side of table shows the data re-calculated using standard months of ~30.4 days. The use of standard months shifts few subjects to an earlier or later interval but maintains the general findings. There were 450–500 births per month in 1967 and 1968, except in February 1968 in which there were somewhat fewer, corresponding to a decrease in conceptions in June 1967, i.e. during the war and soon after, or an increased fetal loss. The raw data suggest a two- to three-fold excess of schizophrenia in the cohort born in January 1968, whose mothers would have been in the second month of pregnancy in June 1967. There was a similar excess of other disorders in the cohort born a month earlier, who would have been in the third month of intrauterine life in June 1967. There were somewhat fewer cases of schizophrenia than expected in the cohort born in August 1967 (exposed in the seventh month), and somewhat more of other disorders in the cohort born in June 1967 (exposed in the final month or at birth). There was no unusual incidence of schizophrenia or other conditions among offspring conceived in the three months after the war, or in those born in the three months before it.

**Table 1:** Numbers of offspring born (N), cases of schizophrenia (Schiz) and other psychiatric disorders (Other psych) in the months surrounding June 1967, by month of birth and estimated stage of life during the war.

**Estimated stage of life during war of June 1967**	**Date of birth**	**Data based on calendar month of birth**			**Data based on standard month of birth**		
		N	Schiz	Other psych	N	Schiz	Other psych
							
**Before conception (months)**							
-3	May 1968	505	4	2	505	4	2
-2	Apr 1968	512	2	4	512	2	4
-1	Mar 1968	498	3	5	498	3	5
							
**Gestational age (months)**							
1	Feb 1968	410	4	3	420	5	3
2	Jan 1968	486	10	1	493	9	3
3	Dec 1967	501	3	11	484	3	9
4	Nov 1967	443	4	1	443	4	1
5	Oct 1967	470	4	4	470	4	4
6	Sept 1967	466	4	3	466	4	3
7	Aug 1967	488	1	6	488	1	6
8	Jul 1967	483	4	5	460	4	5
9	Jun 1967	480	3	10	503	3	10
							
**Post-natal age (months)**							
1	May 1967	512	3	3	496	3	3
2	Apr 1967	424	4	5	424	4	3
3	Mar 1967	471	4	4	476	4	6

Table 2 shows the RRs of schizophrenia by sex, estimated using proportional hazards methods to control for confounding variables; and table 3 shows the same for other psychiatric conditions. The nine birth cohorts exposed in each month of intrauterine life are compared, simultaneously, with the remainder of the cohort. The incidence of schizophrenia was more than doubled for offspring who were in the second month of gestation in June 1967. The risk for other psychiatric disorders was also increased for those who were in the third month of pregnancy at that time, confirming the findings in table 1. In both schizophrenia (table 2) and other conditions (table 3) effects observed after the war were stronger in females than in males.

**Table 2:** Numbers of cases, relative risks (RR) and 95% confidence limits (CL) for schizophrenia, by standard month of birth and sex.

**Estimated gestational month in June 1967**	**Date of birth**	**Total, including unknown sex**			**Males**			**Females**		
				
		**Cases**	**RR^1^**	**95% CL^1^**	**Cases**	**RR**	**95% CL^1^**	**Cases**	**RR**	**95% CL^1^**
1	Feb 1968	5	1.09	0.44–2.72	5	2.01	0.81–5.41	0	-	-
2	Jan 1968	9	** 2.28	1.12–4.65	3	1.17	0.36–3.83	6	*** 4.33	1.71–11.0
3	Dec 1967	3	0.76	0.24–2.44	1	0.43	0.06–3.16	2	1.23	0.29–5.28
4	Nov 1967	4	1.07	0.38–3.00	3	1.30	0.40–4.27	1	0.71	0.09–5.34
5	Oct 1967	4	0.82	0.30–2.27	3	1.17	0.36–3.83	1	0.44	0.06–3.22
6	Sept 1967	4	0.99	0.36–2.74	2	0.92	0.22–3.88	2	1.06	0.25–4.50
7	Aug 1967	1	0.26	0.04–1.89	0	-	-	1	0.94	0.12–7.22
8	Jul 1967	4	0.97	0.35–2.67	1	0.45	0.06–3.26	3	1.58	0.47–5.30
9	Jun 1967	3	0.64	0.20–2.03	1	0.35	0.05–2.57	2	1.07	0.25–4.55

Comparison group (all others)	1964–76	602	1	reference	355	1	reference	245	1	reference

1. Adjusted for paternal age (continuous), maternal age (30–34, 35+ versus all others), low social class (father's occupation group 5,6 versus 1–4), years married and month of birth. 2. Additionally adjusted for sex. ** p < .03; *** p < .003.

**Table 3:** Numbers of cases, relative risks (RR) and 95% confidence limits (CL) for other psychiatric conditions, by standard month of birth and sex.

**Estimated gestational month in June 1967**	**Date of birth**	**Total, including unknown sex**			**Males**			**Females**		
				
		**Cases**	**RR^1^**	**95% CL^2^**	**Cases**	**RR**	**95% CL^1^**	**Cases**	**RR**	**95% CL^1^**
1	Feb 1968	3	0.73	0.23–2.34	3	1.11	0.34–3.64	0	-	-
2	Jan 1968	3	0.60	0.19–1.92	2	0.61	0.15–2.52	1	0.57	0.08–4.25
3	Dec 1967	9	** 2.51	1.22–5.17	4	1.82	0.63–5.24	5	*** 3.55	1.31–9.65
4	Nov 1967	1	0.22	0.03–1.61	0	-	-	1	0.51	0.07–3.77
5	Oct 1967	4	1.02	0.37–2.87	1	0.46	0.06–3.39	3	1.76	0.52–5.93
6	Sept 1967	3	0.90	0.28–2.92	1	0.63	0.09–4.71	2	1.14	0.27–4.87
7	Aug 1967	6	1.56	0.67–3.65	3	1.20	0.37–3.91	3	2.25	0.66–7.69
8	Jul 1967	5	1.04	0.42–2.60	4	1.81	0.64–5.13	1	0.38	0.05–2.79
9	Jun 1967	10	* 1.97	1.01–3.85	6	1.89	0.80–4.46	4	2.11	0.73–6.10

Comparison group (all others)	1964–76	633	1	reference	363	1	reference	270	1	reference

1. Adjusted for paternal age (continuous), maternal age (30–34, 35+ versus all others), low social class (father's occupation group 5,6 versus 1–4), years married and month of birth. 2. Additionally adjusted for sex. * p < .05; **p < .02 *** p < .003.

When the intrauterine exposure to the war was classified by trimesters rather than by months, differences were smaller and not significant. Offspring whose mothers were in the first, second and third trimesters during the war showed adjusted RRs, respectively of 1.33 (0.80–2.12), 0.95 (0.53–1.71) and 0.62 (0.30–1.26). For other psychiatric conditions the corresponding RRs were 1.13 (0.67–1.93), 0.68 (0.33–1.38) and 1.49 (0.94–2.34).

We conducted a post-hoc analysis of schizophrenia comparing the cohort presumed to have been in the second month of gestation at the time of the June 1967 war versus all others (data not tabulated). The excess incidence was observed more in offspring of fathers aged < 30 (RR = 3.7, 1.4–10.0, based on 5 cases in 227 offspring versus 194/39,602) than in offspring of fathers aged 30+ (1.5, 0.5–4.3, based on 4/405 vs 284/47,691); more in association with the two lower social classes (4.3, 1.7–11, 7/210 vs 186/23,687) than in the four higher social classes (0.9, 0.2–3.6, 2/427 vs 298/64,505) and more in those whose mothers had 0–8 years of education (3.2, 1.4–7.5, 7/254 vs 209/29,661) than in those with 9+ years of education (0/331 vs 193/52,504). Residence in census tracts bordering the 1948 border was not a general risk factor for schizophrenia in the whole cohort (RR = 1.1, 0.6–2.0 based on 9 cases in 1,125 offspring); however, this residential area showed an excess risk of schizophrenia in those who were in the second month of intrauterine life during the war (RR = 33, 2.7–400 based on 2 cases in 7 offspring versus a reference group of 7 cases in 1116 offspring).

We did not extend the study to cover the 1973 war because insufficient time had passed for the accrual of cases of schizophrenia. A preliminary analysis, however, suggested consistent findings; there was an increased incidence of schizophrenia in offspring of mothers who had been in the second month of gestation in October 1973 (RR = 1.4, 0.6–3.5) and of other conditions in those who were in the third month (RR = 1.9, 0.9–4.1).

We questioned whether the excess risk of schizophrenia after the June 1967 war might be associated with changes in the distribution of birth weight or gestational age, since maternal stress is believed to contribute to pre-term birth [37] and low birth weight has been associated with schizophrenia [38]. In this cohort, there was a weak and non-significant relationship between low birth weight (< 2500 g) and schizophrenia (RR = 1.2, 0.9–1.7) after controlling for confounding variables. In the cohort estimated to have been in the second month of intrauterine life in June 1967 we observed an unusually low proportion of low birth weight offspring (3.2% versus 6.3% expected). Controlling for low birth weight, however, increased the RR for schizophrenia and narrowed its confidence interval. Similarly, controlling for low birth weight led to a small increase in the RR of other psychiatric disorders, for those in the third month of gestation during the war.

**Figure 1:**
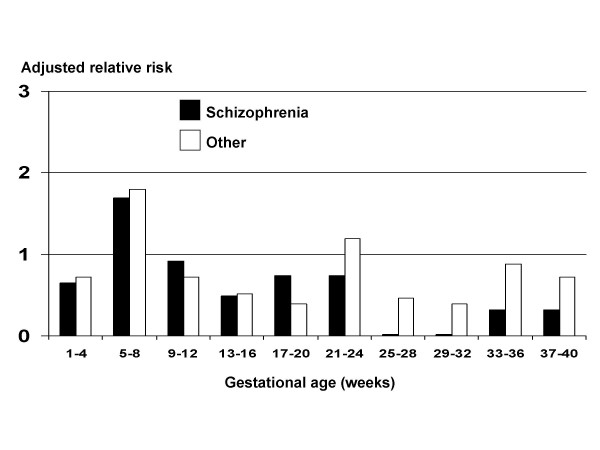
**Relative risks of schizophrenia and other psychiatric conditions in offspring born to mothers pregnant during war of June 1967.** Sub-cohort of 9,519 offspring with information on gestational age.

To corroborate the findings from the whole cohort, we studied the sub-cohort (N = 11,467) with data on of last menstrual date and/or expected date of delivery. From the 11,040 traced live-born offspring, we excluded 1,421 with uncertain gestational age (day of month set at "0" or "10"), leaving 9,519 available for analysis, with 91 cases of schizophrenia. Figure 1 shows adjusted relative risks of schizophrenia according to estimated gestational age in the war. There were wide confidence limits so that none of the RRs were statistically significant; however, they confirm an increased incidence of schizophrenia and other conditions in offspring whose mothers were stressed in early pregnancy (specifically in the second month) and fewer than expected cases of schizophrenia following stress in later pregnancy. This conclusion was unchanged after controlling for pre-term births (i.e. gestations of less than 37 weeks) or for low birth weight (< 2500 g). The figure also suggests an excess incidence of other conditions in early pregnancy.

Conclusions for this study were not altered further adjustment for ethnic groups or by restricting the cohort to singleton births or to Jews.

## Discussion

This pilot study shows that a time-limited threat likely to have caused severe anxiety in pregnant women was associated with an altered incidence of schizophrenia in the offspring. Although based on small numbers, our results are consistent with the studies from Finland [9], the Netherlands [11] and Denmark [10] that pointed to early pregnancy as a period of vulnerability to stress, for schizophrenia. Our prior hypothesis, building on knowledge from teratology, was that fetal effects of maternal stress, if found, would be limited to a short window of time, though we did not specify in advance which would be the most vulnerable month.

This study's finding of sex differences is also consistent with previous publications linking schizophrenia to famine in the Netherlands and China. The Dutch famine of 1945 [11] was followed by a significantly increased incidence of schizophrenia in females born after maternal starvation during the first trimester, or in both first and second trimesters, but was not significantly altered by starvation in the second or third trimesters. In males, schizophrenia was not significantly related to the Dutch famine; however, there was a subtle decrease in its incidence following maternal starvation during the second trimester [11] a subsequent study showed those males to be suffering more from unipolar affective disorders [39]. The Chinese famine of 1959–61 was followed by an increased incidence of schizophrenia among adults born in those years, with a decreased proportion of males among cases [12], suggesting that the increase might have been more severe among females, or that females vulnerable to schizophrenia might have survived better than similarly vulnerable males. After the 9/11 terrorist attack in New York, there was evidence for a selective loss (i.e. spontaneous abortion) of male fetuses. This was observed not only in New York [40] but also in California [41], so it can be attributed to maternal stress alone, rather than to a toxic environment. Thus, the sex difference in schizophrenia observed in the Jerusalem, Dutch and Chinese cohorts might be explained either by greater vulnerability of females to stress (or starvation), or by a greater mortality of vulnerable males.

The most convincing evidence that schizophrenia can follow intrauterine stress derives from investigations of mothers bereaved in pregnancy. Bereavement is a stressor that is not normally accompanied by famine or a toxic environment. In a national register in Finland, Huttunen and Niskanen compared the offspring of 167 mothers whose husbands died during their pregnancy versus those of 168 similarly bereaved after delivery [9]. There was an excess incidence of schizophrenia in the first group, especially when the husband's death occurred early in gestation. A much larger study was reported recently by Khashan et al using the national registry in Denmark; with a follow-up of 10–32 years they observed 1893 cases of schizophrenia (0.3% of the cohort) [10]. The death of one or more of the mother's first degree relatives was linked to a statistically significant excess of schizophrenia, with RR = 1.6–1.7, if the death occurred in the first trimester. Furthermore, the Danish study showed a non-significant reduction in schizophrenia if the death occurred in the third trimester; our findings are similar. Neither the Finnish nor the Danish studies separated males from females; future studies should do this and attempt to determine specificity of timing in gestation, more narrowly than by trimester.

Strengths of the Jerusalem study include its prospective design, a follow-up of 22–34 years, an unusually circumscribed psychological stressor and our ability to stratify by sex, consider season and control for potential confounders such as paternal age. Use of a previously defined cohort and of Israel's national Psychiatric Registry assures an unbiased ascertainment of "exposure" relative to "outcome", without the need to rely on maternal recall or question the diagnosis; diagnoses of psychoses have been recently validated [30]. An additional advantage is the availability of a sub-cohort with information of gestational age, which, though small, permits us to corroborate the findings based on the entire cohort. A limitation is the small numbers. Also, in common with most other previous studies, ours suffers from the drawback from the absence of information on length of gestation. If maternal stress were to lead to an excess of pre-term births in vulnerable pregnancies, and if these were the same individuals vulnerable to schizophrenia in the future, then there might be a systematic bias in the estimation of gestational age in the affected cases; gestations might have been more advanced that we can estimate. Because of this, our results should be regarded as "hypothesis generating" rather than proof that the moment of greatest vulnerability to schizophrenia occurs in the second, rather than any other, gestational month. Others limitations are that we cannot assess in this pilot study which of the "other" diagnostic categories were related to the June 1967 war; and because the cohort has not yet reached middle age, not all potential cases of schizophrenia have become known. We do not know how individual mothers experienced the war, what was their previous or ancestral response to stress, or whether they were injured or bereaved in June 1967 and might have been medicated; and we do not yet know whether the risk of schizophrenia in the offspring might have been modified by events in post-natal life.

There is overwhelming experimental evidence that stress to pregnant mammals alters neurodevelopment in the offspring and affects their subsequent behavior, both in childhood and as adults [42-45]. Furthermore, maternal stress permanently programs metabolic and cardiovascular physiology [1] in the offspring, with consequences for longevity. Almost all of the experimental work, however, has applied stress in mid- or later pregnancy. Mechanisms are unclear and the nature and reasons for sex differences even more so, but experimental and human data suggest that both the neurobehavioral and metabolic effects seem to depend on the same mechanisms, or similar ones [8,46]. These mechanisms involve perturbation of the HPA axis, glucocorticoids and catecholamines [44,46,47], changes in the "set points" for physiologic feedback loops (e.g. leptin, insulin, cortisol) [48] and alterations in ways individuals sense and respond, in the future, to dynamic changes in the environment [8]. Current thinking is that the signal is achieved by epigenetic changes (e.g. methylation at CpG islands, or modification of histones) that alter gene expression and that genetic variability may alter vulnerability (e.g. deficiencies in genes affecting 1-carbon metabolism (methylation)); if so, genetically vulnerable individuals might be protected by modification of the diet at the time of being stressed.

The knowledge that maternal stress affects the fetus has important implications for mental health in a world threatened by acute violence and war. Terrorist acts have stressed whole populations nation-wide, while more locally, hurricanes and earthquakes are recurring stressors in some countries. Smaller communities or individuals can be affected acutely by tornados, episodes of community violence, accidents, bereavement, domestic violence or rape. A significant component of the public burden of mental illness may follow such frights to pregnant women. The subject of intrauterine stress and major psychiatric disorders deserves further scrutiny. Well designed research studies should be aimed at elucidating the pathways to risk and at defining strategies and interventions for prevention.

## Conclusion

This study confirms previous reports of an excess incidence of schizophrenia in offspring born to mothers who experienced stress in early pregnancy. It suggests both sex specificity and a relatively narrow window of vulnerability, in the second month of pregnancy.

## Competing interests

The authors declare that they have no competing interests.

## Authors' contributions

DM conceived and coordinated the studies of schizophrenia and participated in drafting the manuscript. CC, KRK and MCP reviewed literature and participated in drafting the manuscript. SF and DN participated in design and coordination of the study. YF oversaw management of the Jerusalem cohort in Israel. SH conceived the analysis and participated in drafting the manuscript. All authors read and approved the final manuscript.

## Pre-publication history

The pre-publication history for this paper can be accessed here:



## References

[B1] Seckl JR, Holmes MC (2007). Mechanisms of disease: glucocorticoids, their placental metabolism and fetal 'programming' of adult pathophysiology. Nature clinical practice.

[B2] Mandyam CD, Crawford EF, Eisch AJ, Rivier CL, Richardson HN (2008). Stress experienced *in utero* reduces sexual dichotomies in neurogenesis, microenvironment, and cell death in the adult rat hippocampus. Dev Neurobiol.

[B3] Bowman RE, MacLusky NJ, Sarmiento Y, Frankfurt M, Gordon M, Luine VN (2004). Sexually dimorphic effects of prenatal stress on cognition, hormonal responses, and central neurotransmitters. Endocrinology.

[B4] Richardson HN, Zorrilla EP, Mandyam CD, Rivier CL (2006). Exposure to repetitive versus varied stress during prenatal development generates two distinct anxiogenic and neuroendocrine profiles in adulthood. Endocrinology.

[B5] Pallares ME, Scacchi Bernasconi PA, Feleder C, Cutrera RA (2007). Effects of prenatal stress on motor performance and anxiety behavior in Swiss mice. Physiol Behav.

[B6] Mueller BR, Bale TL (2007). Early prenatal stress impact on coping strategies and learning performance is sex dependent. Physiol Behav.

[B7] Talge NM, Neal C, Glover V (2007). Antenatal maternal stress and long-term effects on child neurodevelopment: how and why?. Journal of child psychology and psychiatry, and allied disciplines.

[B8] Phillips DI (2007). Programming of the stress response: a fundamental mechanism underlying the long-term effects of the fetal environment?. Journal of internal medicine.

[B9] Huttunen MO, Niskanen P (1978). Prenatal loss of father and psychiatric disorders. Archives of general psychiatry.

[B10] Khashan AS, Abel KM, McNamee R, Pedersen MG, Webb RT, Baker PN, Kenny LC, Mortensen PB (2008). Higher risk of offspring schizophrenia following antenatal maternal exposure to severe adverse life events. Archives of general psychiatry.

[B11] Susser ES, Lin SP (1992). Schizophrenia after prenatal exposure to the Dutch Hunger Winter of 1944–1945. Archives of general psychiatry.

[B12] St Clair D, Xu M, Wang P, Yu Y, Fang Y, Zhang F, Zheng X, Gu N, Feng G, Sham P (2005). Rates of adult schizophrenia following prenatal exposure to the Chinese famine of 1959–1961. Jama.

[B13] van Os J, Selten JP (1998). Prenatal exposure to maternal stress and subsequent schizophrenia. The May 1940 invasion of The Netherlands. Br J Psychiatry.

[B14] Selten JP, Graaf Y van der, van Duursen R, Gispen-de Wied CC, Kahn RS (1999). Psychotic illness after prenatal exposure to the 1953 Dutch Flood Disaster. Schizophrenia research.

[B15] Watson JB, Mednick SA, Huttunen M, Wang X (1999). Prenatal teratogens and the development of adult mental illness. Development and psychopathology.

[B16] Laplante DP, Barr RG, Brunet A, Galbaud du Fort G, Meaney ML, Saucier JF, Zelazo PR, King S (2004). Stress during pregnancy affects general intellectual and language functioning in human toddlers. Pediatr Res.

[B17] Kinney DK, Miller AM, Crowley DJ, Huang E, Gerber E (2008). Autism prevalence following prenatal exposure to hurricanes and tropical storms in Louisiana. J Autism Dev Disord.

[B18] Schneider ML, Roughton EC, Koehler AJ, Lubach GR (1999). Growth and development following prenatal stress exposure in primates: an examination of ontogenetic vulnerability. Child development.

[B19] Morgane PJ, Austin-LaFrance R, Bronzino J, Tonkiss J, Diaz-Cintra S, Cintra L, Kemper T, Galler JR (1993). Prenatal malnutrition and development of the brain. Neuroscience and biobehavioral reviews.

[B20] Harlap S, Davies AM, Deutsch L, Calderon-Margalit R, Manor O, Paltiel O, Tiram E, Yanetz R, Perrin MC, Terry MB (2007). The Jerusalem Perinatal Study cohort, 1964–2005: methods and a review of the main results. Paediatr Perinat Epidemiol.

[B21] Parker RB (1996). Israel-Arab War, 1967.

[B22] "Six-Day War". http://encarta.msn.com/encyclopedia_761570433/Six-Day_War.html.

[B23] Selten JP, Cantor-Graae E, Nahon D, Levav I, Aleman A, Kahn RS (2003). No relationship between risk of schizophrenia and prenatal exposure to stress during the Six-Day War or Yom Kippur War in Israel. Schizophrenia research.

[B24] Davies AM, Prywes R, Tzur B, Weiskopf P, Sterk VV (1969). The Jerusalem Perinatal Study: 1. Design and organization of a continuing, community-based, record-linked survey. Israel J Med Sci.

[B25] Malaspina D, Harlap S, Fennig S, Heiman D, Nahon D, Feldman D, Susser ES (2001). Advancing paternal age and the risk of schizophrenia. Arch Gen Psychiatry.

[B26] Perrin MC, Opler MG, Harlap S, Harkavy-Friedman J, Kleinhaus K, Nahon D, Fennig S, Susser ES, Malaspina D (2007). Tetrachloroethylene exposure and risk of schizophrenia: offspring of dry cleaners in a population birth cohort, preliminary findings. Schizophrenia research.

[B27] Harlap S, Friedlander Y, Barchana M, Calderon R, Deutsch L, Kleinhaus KR, Perrin MC, Tiram E, Yanetz R, Paltiel O (2007). Late fetal death in offspring and subsequent incidence of prostate cancer in fathers: The Jerusalem Perinatal Study cohort. The Prostate.

[B28] Harlap S, Paltiel O, Friedlander Y, Calderon-Margalit R, Deutsch L, Kleinhaus KR, Manor O, Neugut AI, Opler M, Perrin MC (2007). Prostate cancer in fathers with fewer male offspring: the Jerusalem Perinatal Study cohort. Journal of the National Cancer Institute.

[B29] Perrin MC, Terry MB, Kleinhaus K, Deutsch L, Yanetz R, Tiram E, Calderon-Margalit R, Friedlander Y, Paltiel O, Harlap S (2007). Gestational diabetes and the risk of breast cancer among women in the Jerusalem Perinatal Study. Breast Cancer Res Treat.

[B30] Weiser M, Kanyas K, Malaspina D, Harvey PD, Glick I, Goetz D, Karni O, Yakir A, Turetsky N, Fennig S (2005). Sensitivity of ICD-10 diagnosis of psychotic disorders in the Israeli National Hospitalization Registry compared with RDC diagnoses based on SADS-L. Compr Psychiatry.

[B31] Kimhy D, Harlap S, Fennig S, Deutsch L, Draiman BG, Corcoran C, Goetz D, Nahon D, Malaspina D (2006). Maternal household crowding during pregnancy and the offspring's risk of schizophrenia. Schizophrenia research.

[B32] Reichenberg A, Rabinowitz J, Weiser M, Mark M, Kaplan Z, Davidson M (2000). Premorbid functioning in a national population of male twins discordant for psychoses. The American journal of psychiatry.

[B33] Reichenberg A, Weiser M, Rapp MA, Rabinowitz J, Caspi A, Schmeidler J, Knobler HY, Lubin G, Nahon D, Harvey PD (2006). Premorbid intra-individual variability in intellectual performance and risk for schizophrenia: a population-based study. Schizophrenia research.

[B34] Weiser M, Reichenberg A, Grotto I, Yasvitzky R, Rabinowitz J, Lubin G, Nahon D, Knobler HY, Davidson M (2004). Higher rates of cigarette smoking in male adolescents before the onset of schizophrenia: a historical-prospective cohort study. The American journal of psychiatry.

[B35] Cox DR, Oakes D (1984). Analysis of Survival Data.

[B36] Harlap S, Davies AM, Grover NB, Prywes R (1977). The Jerusalem Perinatal study: the first decade 1964–73. Isr J Med Sci.

[B37] Steer P (2005). The epidemiology of preterm labour. BJOG.

[B38] Clarke MC, Harley M, Cannon M (2006). The role of obstetric events in schizophrenia. Schizophr Bull.

[B39] Brown AS, van Os J, Driessens C, Hoek HW, Susser ES (2000). Further evidence of relation between prenatal famine and major affective disorder. The American journal of psychiatry.

[B40] Catalano R, Bruckner T, Marks AR, Eskenazi B (2006). Exogenous shocks to the human sex ratio: the case of September 11, 2001 in New York City. Hum Reprod.

[B41] Catalano R, Bruckner T, Gould J, Eskenazi B, Anderson E (2005). Sex ratios in California following the terrorist attacks of September 11, 2001. Hum Reprod.

[B42] Huizink AC, Mulder EJ, Buitelaar JK (2004). Prenatal stress and risk for psychopathology: specific effects or induction of general susceptibility?. Psychological bulletin.

[B43] Wadhwa PD (2005). Psychoneuroendocrine processes in human pregnancy influence fetal development and health. Psychoneuroendocrinology.

[B44] Austin MP, Leader LR, Reilly N (2005). Prenatal stress, the hypothalamic-pituitary-adrenal axis, and fetal and infant neurobehaviour. Early human development.

[B45] Kapoor A, Dunn E, Kostaki A, Andrews MH, Matthews SG (2006). Fetal programming of hypothalamo-pituitary-adrenal function: prenatal stress and glucocorticoids. The Journal of physiology.

[B46] Drake AJ, Tang JI, Nyirenda MJ (2007). Mechanisms underlying the role of glucocorticoids in the early life programming of adult disease. Clin Sci (Lond).

[B47] de Weerth C, Buitelaar JK (2005). Physiological stress reactivity in human pregnancy – a review. Neuroscience and biobehavioral reviews.

[B48] Weinstock M (2005). The potential influence of maternal stress hormones on development and mental health of the offspring. Brain, behavior, and immunity.

